# Characterization of doping polycaprolactone (PCL) and nano calcium carbonate (NCC) into polystyrene (PS) thermoplastic network

**DOI:** 10.1038/s41598-025-23821-2

**Published:** 2025-11-18

**Authors:** Eslam Syala, Salah F. Abdellah Ali, Esraa Gaber Emam

**Affiliations:** 1 Department of Materials Science, Institute of Graduate Studies and Researches (IGSR), Alexandria University, 163 Horreya Avenue, Shatby, 21526 Alexandria, Egypt; 2 Chemistry Department, College of Science, Al-Jouf University, 2014 Sakaka, Saudi Arabia

**Keywords:** Polystyrene, PCL, Nano calcium carbonate, Biodegradation, Blends, Engineering, Environmental sciences, Materials science

## Abstract

A series of Polystyrene (PS)-doped Polycaprolactone (PCL) and nano Calcium Carbonate (NCC) blends were prepared using the injection molding and characterized using various techniques to examine the after-effects of blending. Both the FTIR spectra and thermal characteristics proved physical interaction between the ingredients. The tensile strength and Young`s modulus (concerning the mechanical properties) decreased from 40.30 ± 0.852 to 32.78 MPa ± 0.99 and from 3208 ± 45.00 to 2807 MPa ± 30.23 ‘respectively’ as a consequence of NCC agglomeration with increasing its content and the inter-diffusion of lower Young`s modulus PCL within the matrix’s chains. On the other side, the plasticizing action of PCL was responsible for increasing the elongation from 2.0 ± 0.01 to 2.9% ±0.04 and the reduction in the flexural strength from 75.00 ± 0.93 to 47.70 MPa ± 1.45. The biodegradation evaluation readings suggested that microorganisms consumed the PCL, which was responsible for the total weight loss, after 4 months of burial in the soil, and deteriorating the blend’s properties. These results were supported by applying both SEM imaging and mechanical properties testing before and after the degradation.

**Supplementary Information:**

The online version contains supplementary material available at 10.1038/s41598-025-23821-2.

## Introduction

The usage of plastic and plastic products is now increasing day by day. This growth generates an increasing waste stream of plastics in the land, soil, marine environment, etc. About 30% of plastics are used as packaging material throughout the world. Almost all the plastic products are derived from petroleum-based products, which increases the risks of these waste materials and leads to deep environmental and managerial problems because of their non-degradable nature^1^. The main key to getting over these issues is to produce biodegradable polymers as a waste disposal solution. Generally, plastics subjected to biodegradation are decomposed through different steps to produce carbon residuals, CO_2_, H_2_O, and/or CH_4_^2^. Among the aromatic petroleum-based plastics, Polystyrene (PS) is used for a broad range of packaging and building assemblies. Its yearly production reaches approximately 33 million tons, representing around 7% of the entire global plastic production. Most of its residuals are wasted without recycling. PS is generally considered to be resistant to biodegradation because of its recalcitrant macromolecular structure^3^. To enhance its degradation properties by the action of environmental microorganisms, it is preferable to insert a biodegradable artificial aliphatic polyester like polycaprolactone (PCL) in controlled percentages into the PS matrix^4^. The earlier research demonstrated that the addition of PCL accelerates the biodegradation rates, besides its ductility^5,6^. On the other hand and based on their distinct physical, chemical, and mechanical characteristics, nanoparticles of calcium carbonate (NCC) can be merged into the polymer’s blends. They can be added as commercially available, inexpensive fillers participating in enhancing the thermal stability, compatibilization, mechanical, and morphological properties of the polymeric matrix on the basis of their satisfying dispersion throughout the matrix^4^. Particularly, the insertion of NCC can promote thermal conductivity, stiffness, and dimensional stability, reduce the injection molding cycle time, and the relaxation of the inner stresses rather than reducing the final cost of polystyrene^7^. Morais et al. tailored PS/PCL blends with varying weight percentages by twin-screw extrusion. They found that the incorporation of high percentages of PCL deteriorated the mechanical features mainly as a result of the high polarity of PCL’s carbonyl groups^8^. Depending on the important role of molecular weight property on the blend’s compatibility, Mamun et al. studied the influence of PCL’s molecular weight on the versatile properties of the PCL/PS binary blend. The results showed that the interactiveness between the ingredients, besides the crystallinity and thermal stability, are powerfully founded on the molecular weight of PS. Low molecular weight PS (6 K) blends revealed decreased thermal characteristics, while blends with ascending molecular weight PS (650 K) showed promoted thermal stability properties for the blends^9^. Samanta et al. found that the melting temperature was PCL’s content dependent, where the reduction in PCL (wt%) in PS/PCL blends decreased the melting temperature, although the good dispersion of PCL through the matrix^10^. Zha and Fang found that rising the ratios of nano CaCO_3_ in PS/CaCO_3_ blends generally decreased the mechanical characteristics (i.e., impact, tensile strength, and the elongation at break) of the blend^11^. This research aims to explore the after-effect of adding both PCL and nano CaCO_3_ on the mechanical, thermal, physical, water uptake, and biodegradation properties of polystyrene. The novel in this report is preparing an environmentally friendly blend through mixing both PCL and nano CaCO_3_ (in various ratios) and investigating their impacts on the PS matrix, which had not been performed previously to the best of our knowledge, and as indicated after the hard survey. The present research mainly considers promoting the biodegradability of plastics by adding biodegradable materials, like PCL, to them, where these plastics may have limited recycling trials and will eventually be dumped. So, enhancing their biodegradability can achieve the goal of the existing study and finally save both the land and the environment.

## Experimental procedures (Materials, methods, and characterization)

### Materials

General-purpose pure powder polystyrene (GPPS123) was supplied from the local polystyrene production company, E.Styrenics Petrochemical Company, Alexandria, Egypt. Polycaprolactone of average molecular weight (*M*_*wt*_) 80,000, melting point 60 °C, 1.145 g/ml density at 25 °C, and melting flow rate of 2.01–4.03 g/10min was brought from Sigma–Aldrich Co. Ltd., UK. White powder nano calcium carbonate (NCC) grade SN-5300 was obtained from Fujian Sannong Calcium Carbonate Co., Ltd., China, with a particle size of 20–40 nm. To enhance the distribution of the particle throughout the PS matrix, the surface of nano-CaCO_3_ has been treated with stearic acid^4^.

### PS/PCL/NCC blends molding (methods)

The materials were firstly mixed in a robot coupe mixer for 6 min at 3000 rpm speed and dried using a vacuum oven at 50 °C for 24 h before preparation of blends to avert the stuck bubbles in the time of the injection. The injection molding process was executed using an injection molding arrangement type (Arburg allRounder420C-Golden Edition, Germany), single screw. The injection molding was achieved at a temperature profile feeder: T1:210 °C, T2: 220 °C, T3:220 °C, T4:220 °C, and T5:215 °C, screw speed: 90 cm/s, injection press: 1200 bar, cooling time: 60 s, and mold temperature: 45 °C processing conditions. The samples were blended and coded according to proportions and designations in Table 1.

### Characterization of the blends

#### Fourier transform infrared analysis (FTIR)

The possible interactions between the functional groups of the components were discovered by FTIR. Infrared absorption spectra of the blends were recorded using an FTIR spectrophotometer (PerkinElmer-Spotlight 400, USA), with a resolution of 2 cm^− 1^ within the wavelength range 350–4400 cm^− 1^.

#### Thermal characteristics

The Differential Scanning Calorimetry (DSC) test was implemented employing Q1000 TA-Instrument Perkin Elmer as per ASTM D3418-15^12^ to determine the glass transition temperature (*Tg*) of each sample by placing about 8 mg of each sample in an aluminum capsule and heating from 40 to 200 °C with a heating rate of 10 °C/min.

#### Morphology studying

JOEL instrument (JSM-5300) Scanning Electron Microscopy (SEM) was utilized to explore the surface morphology of the as-prepared blends at an accelerated voltage of 25 kV. The samples were fractured, mounted, and sputter-coated with a thin gold coating layer before imaging.

### Mechanical properties testing

#### Tensile properties

The tensile test is applied to detect the tensile strength, yield zone, yield strength, and additional tensile characteristics. It is more sensitive to the nature of the matrix and the interfacial interaction between the different components of the blend^13^. The test was executed following ASTM D638-14^14^ at room temperature, applying a universal testing machine with load cell 10 KN (Zwick /Z010, Germany) at a crosshead speed of 5 mm/min. The specimens were dumbbell-shaped following ISO R527-2^15^ and tested five times for more accuracy.

#### Charpy impact test

Impact examination evaluates the absorbed energy during a specified impact of the sample when a standard weight strikes it at a given speed. Impact strength of PS/PCL/NCC blends was investigated using a pendulum impact tester HIT25P made by Zwick with a 2 J pendulum as per ISO 179/1eU^16^. The test was repeated three times for accuracy purposes.

#### Flexural test

This test expresses the mandatory force to flex a specimen as a consequence of the applied force. The flexural test of the samples was carried out in line with ISO R178^17^ at a speed of 2 mm/min by employing the load cell 10 KN (Zwick /Z010, Germany). For more accuracy, the test was repeated three times, and the average was considered. Furthermore, ANOVA statistical analysis was employed to compare the data of the three sub-groups of blends, besides the neat matrix, with all of the other samples.

### Biodegradation of the blends

For investigating the biodegradability of the prepared series, the samples were sliced into circles with a radius (r) = 2 mm and 4 mm thickness and dried at 50 °C in a vacuum oven, then implanted in soil mud. This mud included different types of Bacillus bacteria of the genus (Bacillus spp). Further bacteria belonging to the genus (Corynebacterium spp), fungi such as (Pythium sp), and (Rhizoctonia solani) with a pH of 7.41, as stated in the report issued by the Faculty of Agriculture, Alexandria University, Egypt. The temperature was kept at room temperature (25 ± 2) °C with the lowest air flow rate. The degradation ratio was determined ascribed to the ratio of weight loss. The samples were kept moisturized in the mud by continual water spraying. The buried samples were preserved for a month in these conditions. After this month, they were taken out from the mud, flushed with distilled water, dried for 24 h in a vacuum oven at 50 °C, and then weighed before putting them back into the soil mud again. This procedure was followed at the end of every month. The weight loss ratio was measured by Eq. (1)

1 $$\:\text{Weight\:Loss\:}\text{(}\text{\%}\text{)\:}\text{=}\frac{\left({\text{W}}_{\text{i}}\text{-}{\text{W}}_{\text{f}}\right)}{{\text{W}}_{\text{i}}}$$

where $\:{W}_{i}\:$and $\:{W}_{f}$ are the weights previous to and after soil burying, successively. The SEM imaging technique was employed again to examine the morphology alteration of the sample’s surface after the burial in mud for 4 months. The examination was carried out in conformity with ASTM D5988–1818 with three samples for each concentration for more accuracy.

### Water absorption experimentation

This examination is applied to observe the amount of absorbed water following definite conditions. The water absorption test of samples was executed in line with ASTM D570–9819. The specimens of the blends were submerged in distilled water and maintained at room temperature for 9 weeks. The water uptake was measured by weighing the samples at regular intervals according to Eq. (2).

2 $$Water~Absorption\% ~ = \frac{{\left( {M_{t} - M_{0} } \right)}}{{M_{0} }}~ \times 100$$

where $\:{\text{M}}_{\text{0}}$ and $\:{\text{M}}_{\text{t}}$ reflect the dry original weight and the weight after immersion time in water, respectively.

## Results and discussion

### Structural analysis (FTIR)

**Fig. 1:**
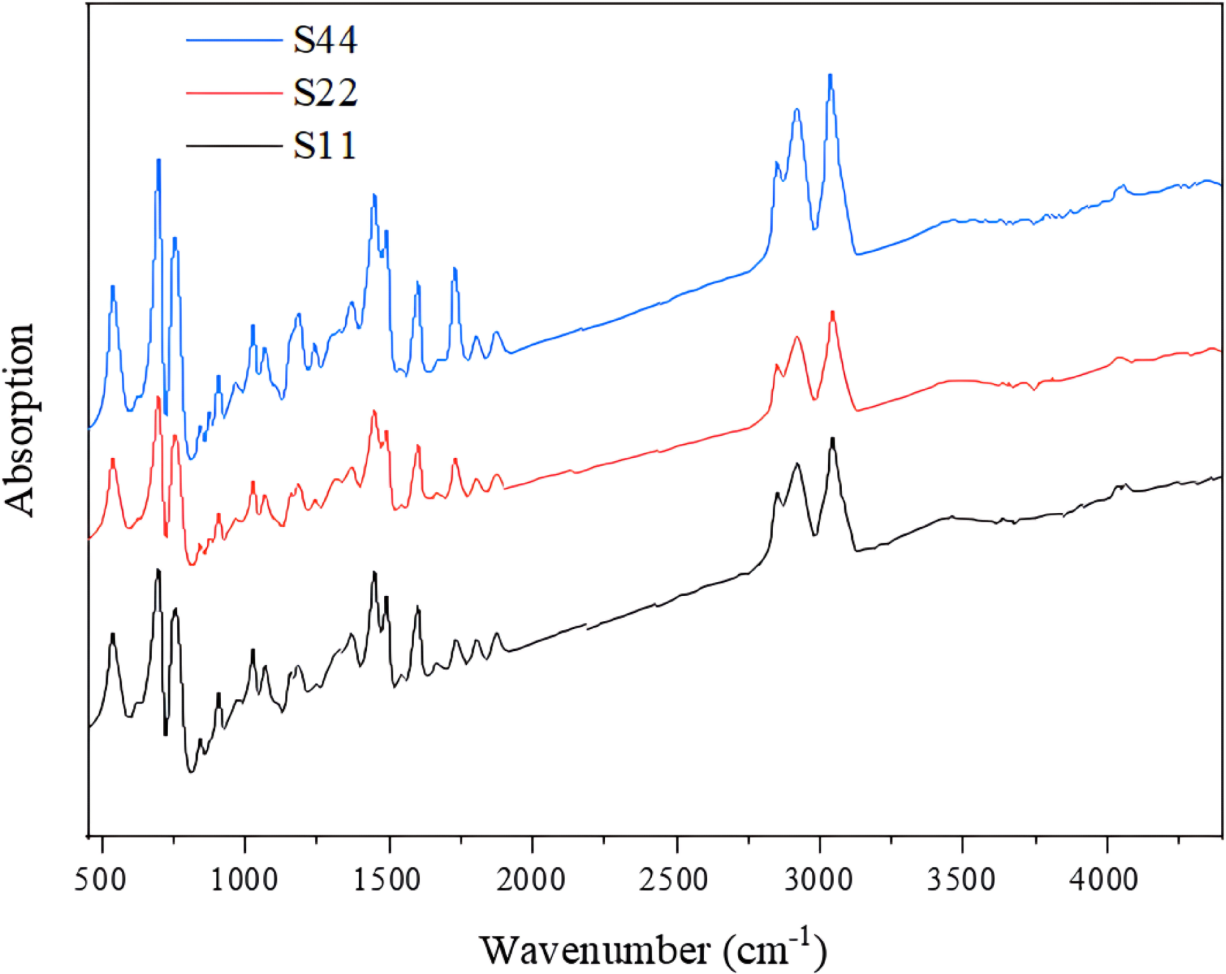
FTIR absorption spectra of S11, S22, and S44 blends as representatives of the prepared series.

Both Figure. 1 and Table S1 expose the FTIR peak assignments of the as-prepared blends. The peaks, which are noted at 1602 and 1492 cm^− 1^, are related to the existence of a benzene ring attached to the carbon atom in the chemical structure of PS. The peak at 1298 cm^− 1^ is in reference to the C–C backbone and C–O stretching patterns in the crystalline PCL. The broad absorption peak at 3455 cm^‒ 1^ refers to stretching and asymmetric vibration of the O–H bond, which may be ascribed to the appearance of absorbed water. The peak at 2362 cm^‒ 1^ is assigned to carbon dioxide in the ambiance^20,21^. The remaining absorption peaks are detailed in Table S1. The spectra propose that the interaction between PS and PCL is via *n*―π bonding (i.e., (*n*) unshared pair electrons of the carbonyl group in PCL and π electrons of the aromatic ring in PS)^22^. The presence of the IR peaks of single ingredients (i.e., PS, PCL, and NCC) in individual curves in Figure. 1 verifies the existence of each ingredient solely, and hence, there is only physical interaction arose among the constituents, as confirmed also in previous literature^9^.

### The glass transition temperature (Tg)

**Fig. 2:**
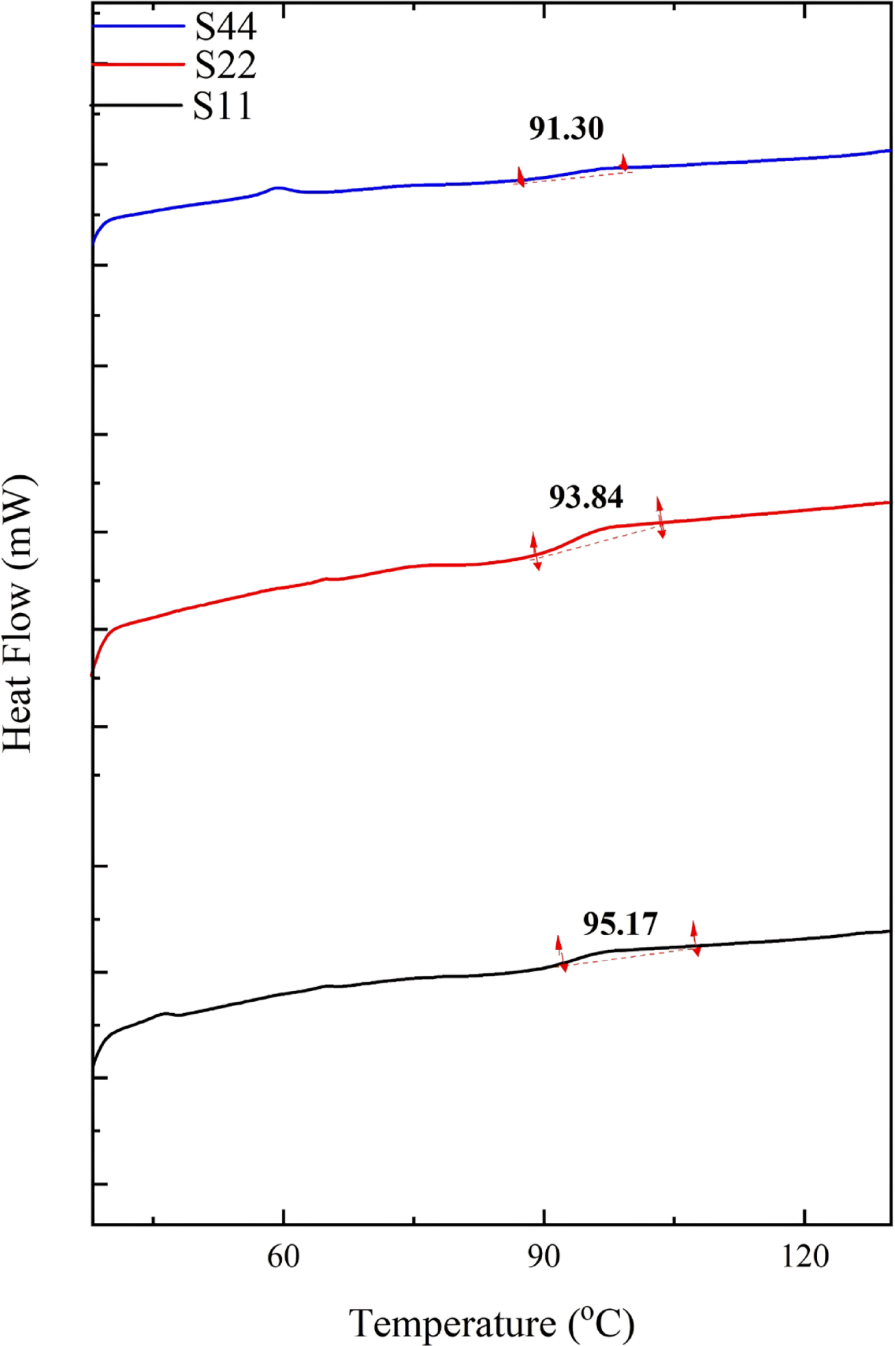
The DSC thermograms of S11, S22, and S44 blends ‘respectively’ as representatives of the prepared blends.

DSC thermograms of the prepared blends are displayed in Figure. 2, while the *Tg* values are recorded in Table 2. Across the system, the *Tg* decreases from 95.32 °C (for S0) to 91.30 °C (for S44). This implies that the prepared blends are thermally and dimensionally stable up to 91.30 °C. The small peak observed at ≈ 60 °C, especially at the highest concentration of PCL, i.e., 4%, is related to the melting of unreacted/uncompatibilized species of PCL. Where PS is highly rigid in nature, by virtue of the existence of rigid groups such as the aromatic rings^23^, reducing its percentage results in a reduction in *Tg*. The incorporation and increasing of PCL, which has a low *Tg* (~ − 60 °C), also participates in lowering the *Tg*. Besides, the continual addition of NCC made it to be aggregated by increasing its content (especially at 4%), which weakens its interfacial adhesion with the PS matrix and possibly increases the embedded free volume and finally reduces the *Tg*^11^. The continuous drooping of *Tg* verifies the poor (physical) interaction between the components of the as-prepared blends^11^, as proved by the FTIR results in the (Structural analysis (FTIR)) section, which revealed that the presence of ester groups existing in PCLs’ backbone enables it to be compatible with the PS matrix through non-covalent weak n―π bonding interaction^22^.

**Table 1:** The compositions and the coding system of the prepared PS/PCL/NCC blends (In the coding system: the first digit = PCL wt.%, while the second digit = NCC wt.%, respectively).

Sample code	PS (wt%)	PCL (wt%)	Nano CaCO_3_ (wt%)
S0	100%	0%	0%
S11	98%	1%	1%
S12	97%	1%	2%
S14	95%	1%	4%
S21	97%	2%	1%
S22	96%	2%	2%
S24	94%	2%	4%
S41	95%	4%	1%
S42	94%	4%	2%
S44	92%	4%	4%

**Table 2:** Mechanical properties and glass transition temperature (*Tg*) of PS-doped PCL/NCC blends.

Sample	Tensile strength at break (MPa)	Youngs modulus (MPa)	Elongation (%) at break	Impact strength (KJ/m²)	Flexure strength (MPa)	Glass transition temperature Tg (^o^C)
S0	40.30 ± 0.852	3208 ± 45.00	2.0 ± 0.01	8.00 ±0.08	75.00 ± 0.93	95.32
S11	42.47 ± 1.15	2997 ± 81.29	2.5 ± 0.03	18.71 ± 0.72	76.60 ± 0.83	95.17
S12	36.85 ± 1.03	2948 ± 33.56	2.7 ± 0.05	16.77 ± 0.42	67.30 ± 0.88	94.66
S14	36.15 ± 0.72	2913 ± 17.00	2.7 ± 0.02	16.26 ± 0.36	64.70 ± 1.36	94.35
S21	36.62 ± 0.95	2918 ± 17.38	2.7 ± 0.08	16.69 ± 0.74	63.40 ± 1.10	94.25
S22	35.99 ± 0.83	2894 ± 97.90	2.7 ± 0.05	15.80 ± 0.70	62.60 ± 1.70	93.84
S24	35.63 ± 1.01	2871 ± 61.23	2.7 ± 0.05	14.59 ± 0.82	62.50 ± 1.97	93.00
S41	35.13 ± 0.90	2854 ± 83.67	2.7 ± 0.05	16.56 ± 0.92	58.50 ± 2.68	92.93
S42	35.02 ± 0.86	2842 ± 72.33	2.8 ± 0.07	14.40 ± 0.56	57.60 ± 1.60	92.46
S44	32.78 ± 0.99	2807 ± 30.23	2.9 ± 0.04	14.10 ± 0.80	47.70 ± 1.45	91.30
F (P)	45.46 (3.21453 × 10^− 18*^)	45.42 (0.000158^*^)	21.31 (0.00133^*^)	14.23 (0.00389^*^)	6.379 (0.026942^*^)	

F for the ANOVA test

p-value for comparison between the various studied groups of blends.

*Statistically significant at p ≤ 0.05

### Phase morphology of the neat PS matrix and its blends

**Fig. 3:**
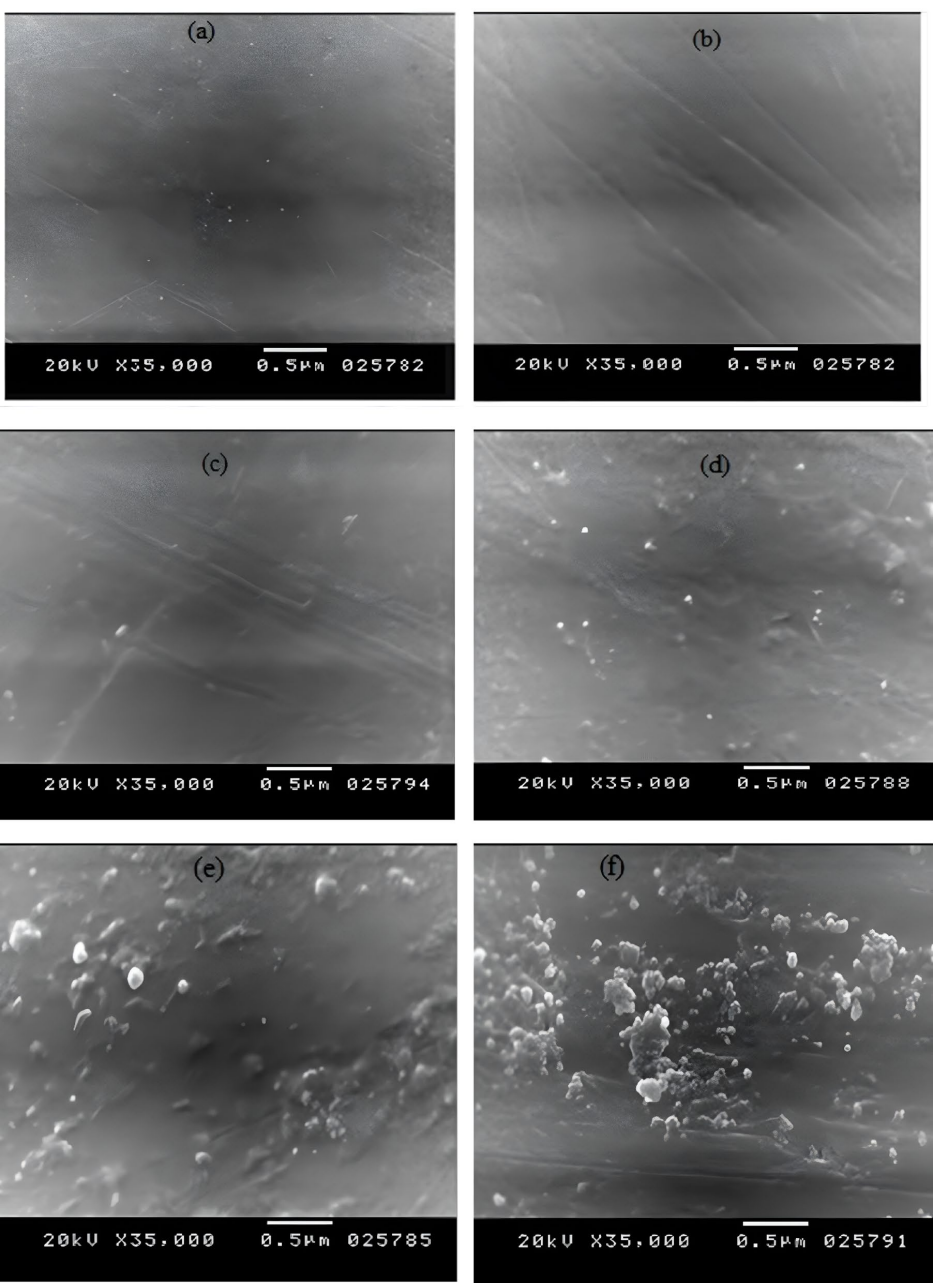

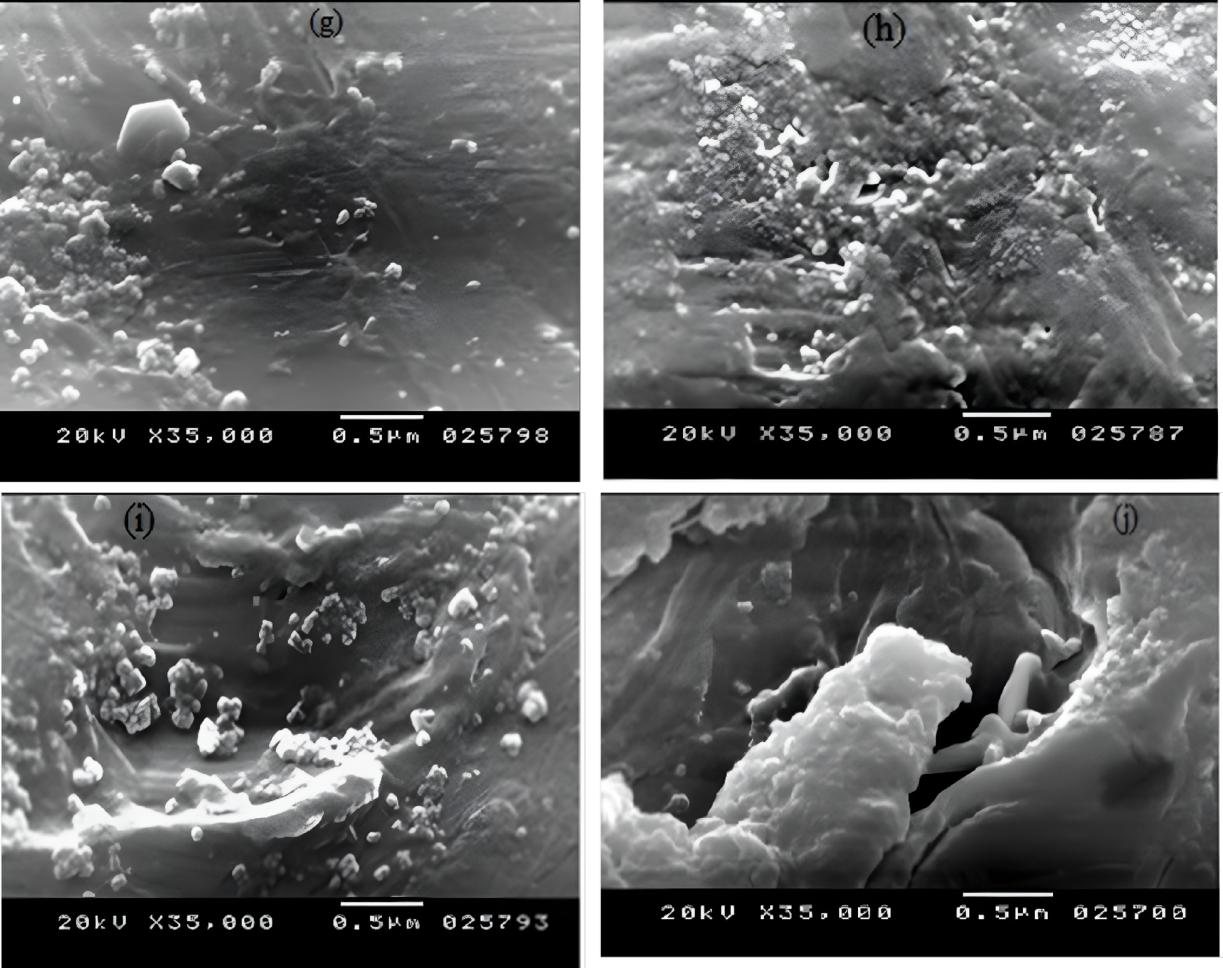
SEM micrographs of (**a**) neat PS, (**b**) S11, (**c**) S21, (**d**) S41, (**e**) S12, **(f**) S22, (**g**) S42, (**h**) S14, (**i**) S24, and (**j**) S44 blends, respectively.

SEM images of the unalloyed PS and the as-processed series are depicted in Figure. 3a-j. The pure PS has a smooth, homogenous, uniform fractured cross-sectional area as in Figure. 3a. Figure. 3b–d detects the best diffusion of NCC in the PS matrix across the different blends, although its cross-sectional area is not as smooth as that of the neat PS; however, it shows no agglomeration. With rising the NCC content, the dispersion becomes a severe problem, and the interval between the nanoparticles becomes smaller, leading to a decline in the dispersion. Attributed to the tendency of NCC to agglomerate, many cavities between the particles and the matrix were observed in the Figure. 3e–j. The formation of these agglomerates could be ascribed to the insufficient interfacial consistency between the hydrophilic NCC and the hydrophobic PS matrix^25^. This is consistent with the conclusions derived from the FTIR and DSC results.

### Mechanical properties

#### Tensile strength at break

Tensile strength is the capacity to withstand deformation or breaking by tensile stress, and it is expressed in terms of force per unit area. Tensile strength of the blends under study improved from 40.3 MPa ± 0.852 (for S0) to 42.47 MPa ± 1.15 (for S11) at 1 wt% of NCC, as seen in Table 2. This strength enhancement can be attributed to the good dispersion of nano CaCO_3_ throughout the matrix at low addition percentages, resulting in a greater strengthening impact and improved interfacial adhesion within the matrix. This yields a higher reinforcement effect and acts as a compatibilizing agent. The continuous loading of NCC gradually decreases the tensile strength from 42.47 MPa ± 1.15 (for S11) to 32.78 MPa ± 0.99 (for S44). This decrease is a consequence of the agglomeration of NCC particles at larger contents that hinders chain mobility and thus stress movement as a result of the poor filler–polymer interaction and finally decreases the tensile strength at break^25^. The current decreasing behavior of the tensile strength with increasing NCC agrees with what has been achieved by previous studies^4^. Another possible reason for these decreases is the presence of voids within the structure, as in the present case, which is compatible with a previous study that used NCC as a filler^26^. One more probable motive for the decrease in tensile strength is the interdiffusion of PCL into the chain structure, reducing the contact and bonding between PS chains (acting as a spacer). Also, polarity and thus compatibility are two important parameters that should be considered for this decrease in the tensile strength, where PS is a non-polar, hydrophobic polymer, while PCL is polar. Because of this polarity variance, PS and PCL are typically immiscible and form phase-separated blends without strong adhesion between the two phases, leading to inefficient load transfer across the interface, decreasing the measured tensile strength. This behavior also matches prior research that studied the effect of increasing the PCL content on the PS matrix^8^.

#### Young`s modulus (E)

**Fig. 4:**
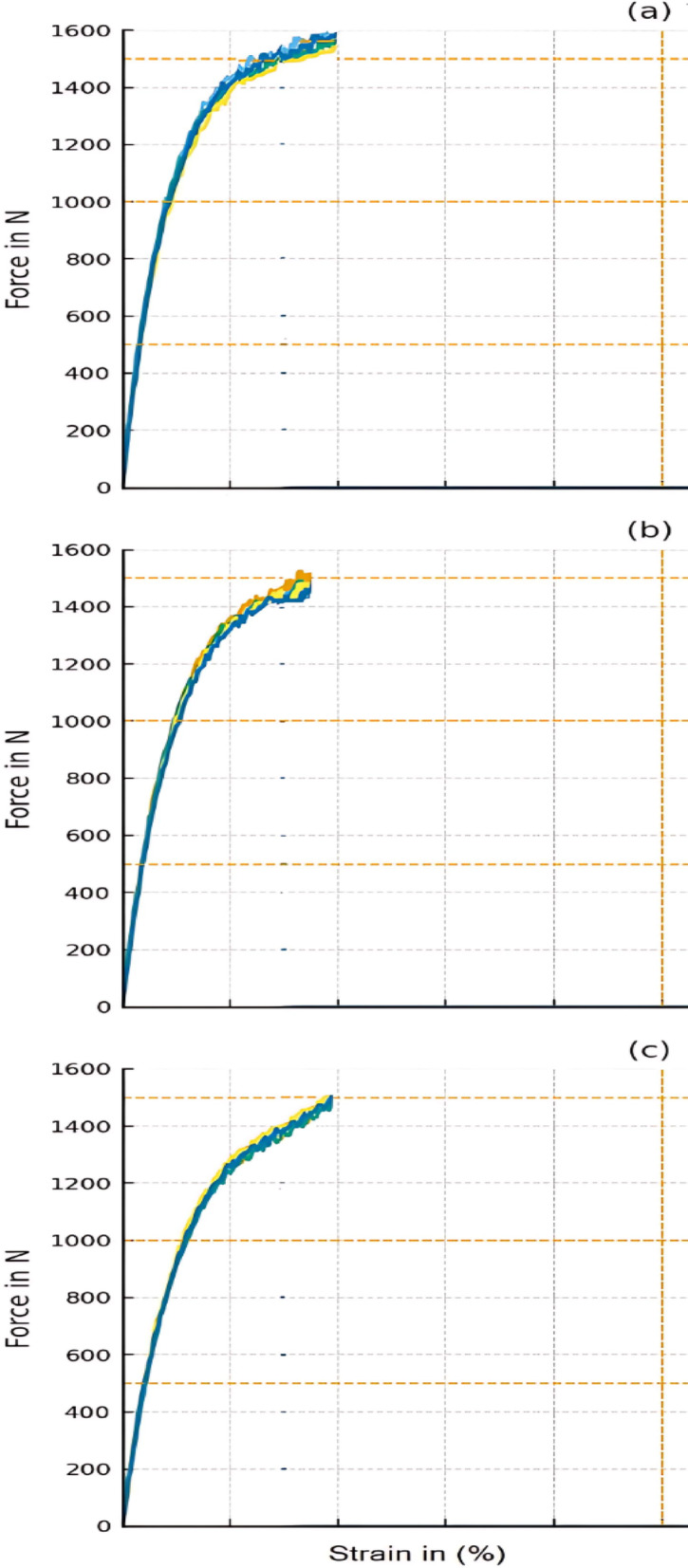
The stress-strain curves of (**a**) S11, (**b**) S22, and (**c**) S44 blends ‘respectively’ as representatives of the prepared blends.

Young’s modulus is an indicator of the load needed to induce a given deformation in the material to the resulted strain. The Young`s modulus of the prepared PS-doped PC/ NCC blends decreases by increasing NCC and PCL contents, as displayed in Table 2; Figure. 4. Decreasing the (E) values is consistent with the general decrease in tensile strength values (Tensile strength at break section). By increasing PCL content, (E) drops from 3208 MPa ± 45.00 (for S0) to 2807 MPa ± 30.23 (for S44). This decreasing trend by growing the PCL content might be ascribable to introducing a lower Young`s modulus polymer (343.9–571.5 (MPa) for PCL) to a higher Young`s modulus (3208 MPa) PS matrix^27^. This observed decreasing trend agrees with what has been concluded by Morais et al. that the addition of PCL to the PS matrix decreased both the Young`s modulus and the tensile strength^8^.

#### Elongation (%) at break

Elongation at break expresses the ability of the material to bear tensile stress without cracking. The elongation of the prepared blends slightly increases with the rise of both PCL and NCC contents in the PS matrix, as revealed by Table 2. By rising the NCC content, the elongation of the as-prepared blends increases from 2% ±0.01 (for S0) to 2.9% ±0.04 (for S44). This may be attributed to the physical interaction between NCC and the PS (as confirmed by FTIR results) that allows the formation of voids in the matrix, which interfere and may be drawn into the deformation areas and thus increase the elongation. Also, growing the PCL content may be another reason for this increase in elongation (%) at break because of two reasons: (1) its flexible nature, compared to the brittle behavior of neat PS, causing a slight plasticization of the PS matrix, reducing the brittleness, as has been concluded by de Souza Morais et al.^28^, (2) its role as a spacer between PS chains. This can promote the use of the prepared blends in applications that require enhanced flexibility and/or elongation, such as packaging applications.

#### Impact strength

The impact strength of the prepared blends is recorded in Table 2. Firstly, it significantly increases along with the initial introduction of PCL and NCC to neat PS from 8 KJ/m^2^ ± 0.08 (for S0) to 18.71 KJ/m^2^ ± 0.72 (for S11). The presence of 1% NCC absorbs the impacted load of energy and protects the blend^29,30^. Also, the particle size of NCC allowed an increment in the interfacial contact area between NCC and PS matrix at low percentages, which led to this rise in the impact values^31^. After that, there is a reduction in the impact strength from 18.71 KJ/m^2^ ± 0.72 (for S11) to 14.10 KJ/m^2^ ± 0.80 (for S44 blend). This sharp decrease is a consequence of the agglomeration that occurred with the addition of more NCC (as confirmed by SEM micrograph analysis), which lowered the interfacial compatibility and created more defect areas, ultimately reducing the impact strength values. Another cause of this decrease is that the inclusion of a semi-crystalline and/or less compatible phase, such as PCL, in the PS matrix can lead to a lowering in molecular mobility and ductility. This causes stress concentration points and restricts plastic deformation (polymer chain mobility near the interface), which generally drops the material’s ability to dissipate energy under impact, thus reducing the measured impact strength. Although the impact strength of the prepared blends decreased with the addition of both PCL and NCC, they still enhanced this property related to the neat PS (14.10 KJ/m^2^ ± 0.80 for S44 compared to 8 KJ/m^2^ ± 0.08 for pure PS), allowing the use of the designed blends in domestic applications, such as toys and device housings, casings, and other internal components in computers.

#### Flexural strength

**Fig. 5:**
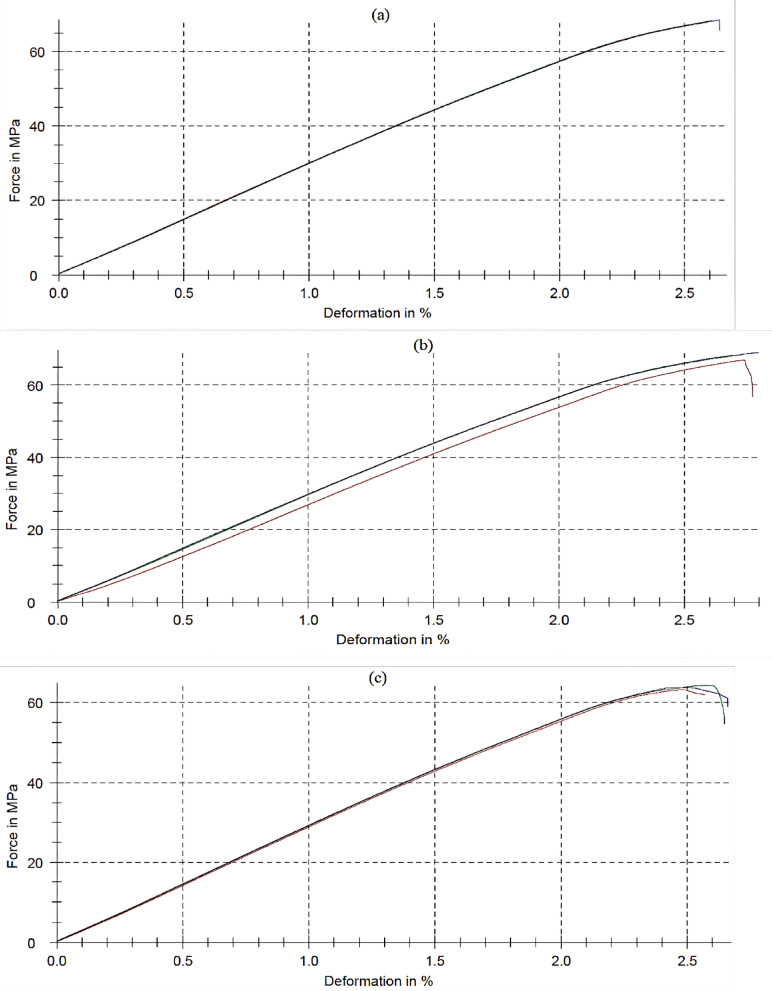
The flexural strength curves of (**a**) S11, (**b**) S22, and (**c**) S44 blends ‘respectively’ as representatives of the prepared blends.

Flexural strength is the stress at bending failure. It is a measure of the material’s resistance to deformation when subject to load. The flexure strength of the PS-doped PC/ NCC blends decreases by increasing the PCL quantity, as shown in both Table 2, Figure. 5. 1% NCC content improved the flexure strength from 75 MPa ± 0.93 (for S0) to 76.6 MPa ± 0.83 (for S11). This improvement is a result of the good dispersion of nanoparticles into the matrix that enhances the interfacial adhesion inside the matrix^29,30^. The subsequent reduction in the flexural strength from 76.6 MPa ± 0.83 (for S11) to 47.70 MPa ± 1.45 (for S44) with the continuous increase of PCL loading in the blends might be attributed to the plasticizing impact of PCL, that have flexible chain units and finally reduces the stiffness of the PS matrix. This interpretation is in agreement with the tensile strength results. Additionally, the PCL has very low flexural strength (23.94 MPa) compared to the flexural strength of the PS (70 MPa)^32-33^, reducing the stiffness and load transfer efficiency in the PS matrix. Table 2 exhibits the ANOVA results comparing the various groups of blends. All p-values are much smaller than 0.05 (e.g., tensile strength, *p* = 3.21 × 10⁻¹⁸, Elongation (%) at break, *p* = 0.00133), indicating statistically significant differences among the studied groups of blends for the investigated properties, confirming the observed variations. These results validate the effect of blend modifications on the material’s mechanical behavior.

### Water absorption experimentation

**Fig. 6:**
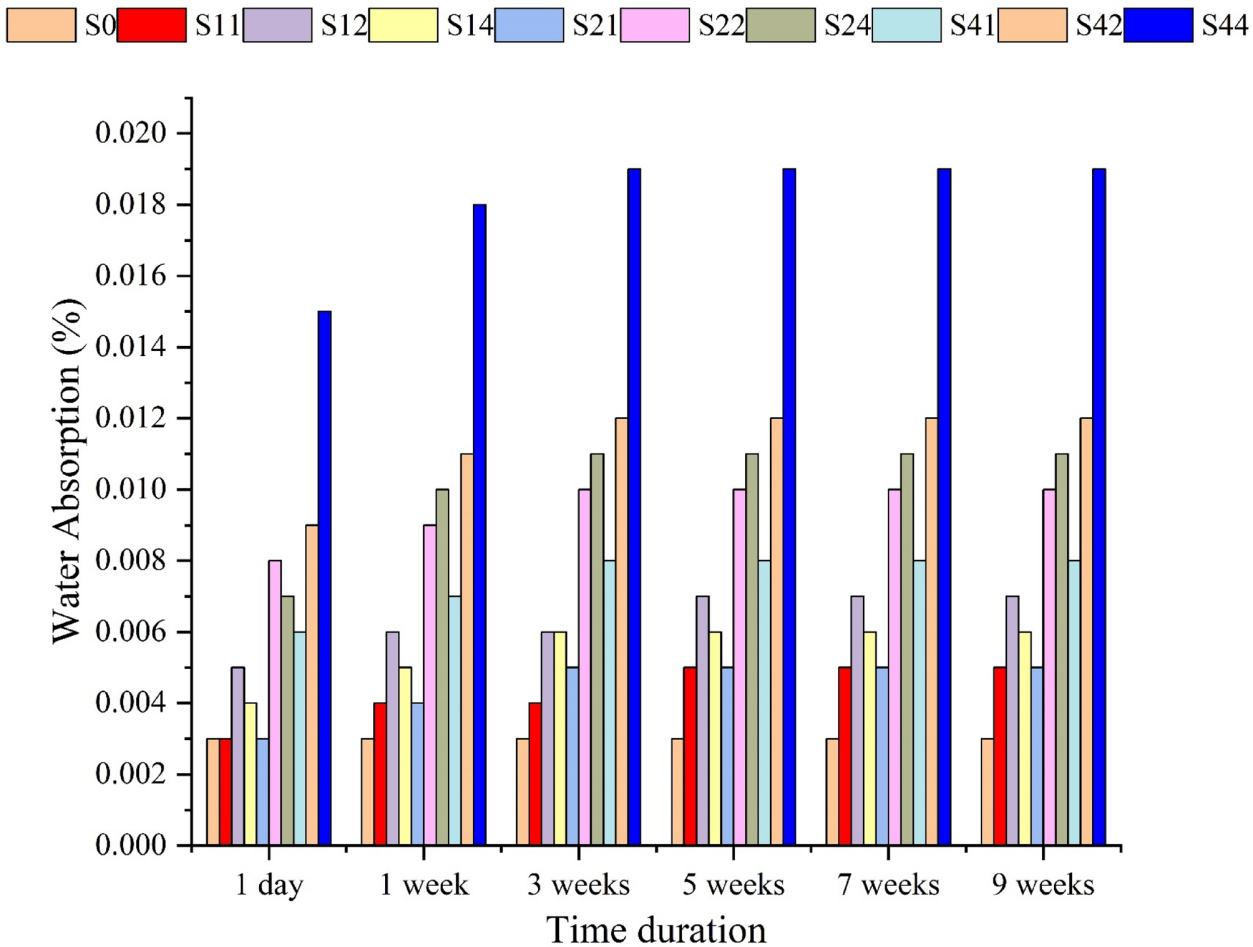
Representation of water absorption data of the prepared PS/PCL/NCC blends.

The water uptake data of the prepared PS/PCL/Nano CaCO_3_ blends after 9 weeks of soaking are displayed in Table 3, Figure. 6. The hydrophobic neat PS absorbed just 0.003% ±0.0001 within the 9 weeks of water submersion, while the water absorption of all PS/ PCL /NCC blends elevated marginally up to 0.019% ±0.0001 (for S44). Only the NCC is responsible for this small increment in water uptake, where PCL is known for its hydrophobic nature and resistance to water, as acknowledged by previous studies^34^. The insertion of NCC and increasing its content (especially at 4% content) promoted the tendency for its nanoparticle’s agglomeration and hence holes and voids formation, allowing more chances for water uptake, as noted from the SEM micrographs (Figure. 3h) where the NCC is the main responsible for the resultant structure, besides its hydrophilic essence, that finally led to this observed minor water absorption rate^35^. However, these minute increases in weights can be ignored compared to increases in other blends^35,36^.

**Table 3:** Water absorption data of the prepared PS/PCL/NCC blends.

Sample	Water absorption (%) / duration ± 0.0001					
	(1 day)	(1week)	(3 weeks)	(5 weeks)	(7 weeks)	(9 weeks)
S0	0.003	0.003	0.003	0.003	0.003	0.003
S11	0.003	0.004	0.004	0.005	0.005	0.005
S12	0.005	0.006	0.006	0.007	0.007	0.007
S14	0.004	0.005	0.006	0.006	0.006	0.006
S21	0.003	0.004	0.005	0.005	0.005	0.005
S22	0.008	0.009	0.010	0.010	0.010	0.010
S24	0.007	0.010	0.011	0.011	0.011	0.011
S41	0.006	0.007	0.008	0.008	0.008	0.008
S42	0.009	0.011	0.012	0.012	0.012	0.012
S44	0.015	0.018	0.019	0.019	0.019	0.019

### Biodegradation test (Soil burial)

**Fig. 7:**
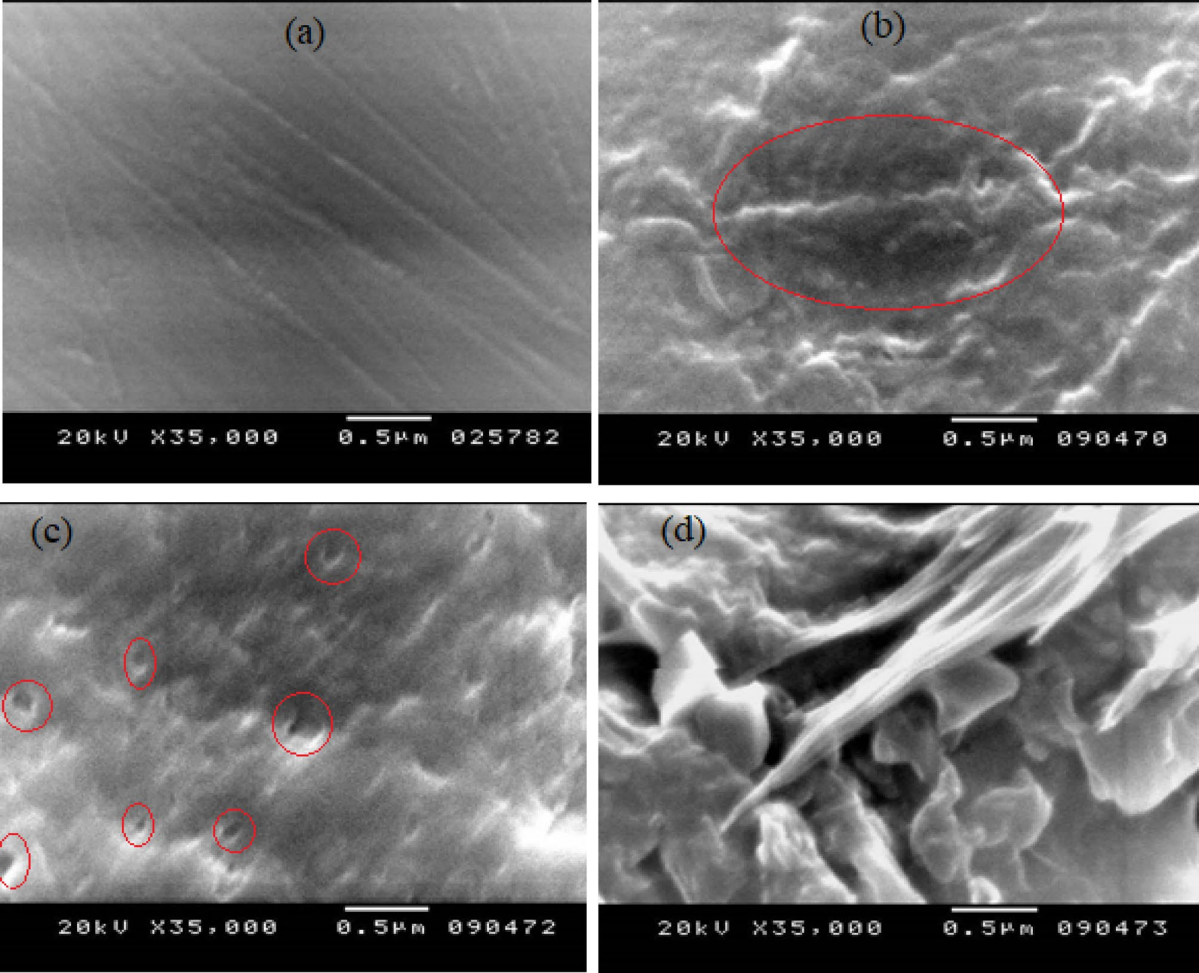
SEM micrographs of (**a**) S0, (**b**) S11, (**c**) S22, (**d**) S44 blends after biodegradation. (The pores resulted from degradation are marked in each picture).

Soil burial is a bio-geophysical experiment that can provide a realistic simulated atmosphere related to soil moisture, temperature, and microorganisms. The weight loss data of the prepared PS/PCL/NCC blends after interment in the mud for four months are tabulated in Table 4. The neat PS (S0) shows that there is no observed weight dropping even at the end of the 4 months. Increasing PCL and NCC induced the biodegradability of PS from 0.07% ±0.0001 (for S11) to 0.67% ±0.0001 (for S44). Increasing weight loss over time indicates that the samples are continuously degrading, suggesting that the microorganisms consume PCL (biodegradable polyester) and create pores in the PS matrix. This fact was confirmed in previous research^37^. The PS is degraded by fungal adhesion, and its morphology is affected by the degradation of the PCL. Inside the matrix, the PCL is protected by PS (i.e., PS slows down the degradation speed of the PCL), which drives the overall biodegradation activity and causes the PCL to be less prone to microbial degradation. To evaluate the degrading effect on the PS and its blends, the SEM characterization was performed again. It can be observed in the Figure. 7a that there is no appearance of holes or fungal growth in the pure PS, which may be thanks to the PS’s resistance to microbial attack/growth on its exterior. Figure. 7b-d presents that the shells of S11, S22, and S44 blends have cavities, cracks, and rugged structures. These morphological alterations reflect microbial activity on the matrix’s surface. These morphological modifications are assigned to the degradation that may happen at the instability locations, just like chain folds, chain ends, and the crystal’s edges, where the mobility of the chains is thought to be greater. The degradation of the lamellar edges may cause lamellar thinning and melting at relatively inferior temperatures than the initial crystals^38^. Additionally, the mechanical properties characterization of the prepared PS-doped PCL/ NCC blends was reperformed to evaluate the biodegradation effects on the blends’ performance. The results in Table 5 reveal the deterioration of the mechanical properties with the continuation of biodegradation for 4 months. The tensile strength at break decreased with percentages started from 2.05 to 6.7%; the Young’s modulus decreased with percentages from 1.03 to 7.8%; the elongation (%) at break lowered with ratios from 1.1 to 6.55%; the impact strength decreased with percentages from 1.6 to 6.24, while the flexure strength decreased with percentages ranged from 1.04 to 8.78%. These decreases are a normal consequence of structural changes caused by the action of the microorganisms during the biodegradation.

**Table 4:** Biodegradation results of the prepared PS/PCL/NCC blends.

Sample	Weight loss (%)/duration ± 0.0001		
	1 Month	2 Months	4 Months
S0	0	0	0
S11	0.04	0.06	0.07
S12	0.06	0.09	0.11
S14	0.10	0.15	0.19
S21	0.12	0.16	0.21
S22	0.18	0.20	0.22
S24	0.24	0.26	0.27
S41	0.30	0.35	0.40
S42	0.44	0.50	0.55
S44	0.50	0.60	0.67

**Table 5:** The mechanical properties of PS-doped PCL/ NCC blends after a 4-month biodegradation process.

Sample	Tensile strength at break (MPa) ± 1.03	Young’s Modulus (MPa) ± 53.05	Elongation (%) at break ± 0.066	Impact strength (KJ/m²) ± 0.88	Flexure strength (MPa) ± 3.00
S0	40.00	3200	2.00	8.00	75.00
S11	41.92	2969	2.47	18.41	75.80
S12	36.42	2924	2.67	16.46	66.19
S14	35.69	2868	2.65	15.82	63.42
S21	35.54	2843	2.66	16.19	62.48
S22	34.93	2822	2.65	15.17	60.96
S24	34.27	2801	2.66	13.97	60.94
S41	33.87	2742	2.64	16.04	55.83
S42	33.07	2682	2.62	13.72	54.83
S44	30.61	2589	2.71	13.22	43.51
F (P)	4.48 (0.056331^*^)	26.32 (0.000749^*^)	25.83 (0.000789^*^)	11.33 (0.0006952^*^)	7.04 (0.021568*)

F for the ANOVA test.

p-value for comparison between the various studied groups of blends.

*: Statistically significant at *p* ≤ 0.05.

## Conclusion

The polystyrene (PS) was blended with PCL and nano CaCO_3_ with percentages ranged from 1 to 4 wt% by the injection molding technique. Different tests and evaluation techniques were performed on the blends, and the results were compared with the neat PS matrix. The SEM images revealed the pores spreading and the tendency to agglomeration with the rise in the NCC content. The FTIR spectra analysis proposed that the interaction between the ingredients was only physical via *n*―π bonding, and this fact was confirmed by DSC results interpretation. The agglomeration of NCC particles, with the growing of their percentage, was the main reason for the general reduction of both the tensile strength from 42.8 MPa (for S11) to 32.8 MPa (for S44) and the impact strength from 18.71 KJ/m^2^ (for S11) to 14.10 KJ/m^2^ (for S44 blend). On the other side, rising the plasticizing action of PCL with its higher contents was one of the leading reasons for increasing the elongation of the samples from 2% (for S0) to 2.9% (for S44) and the reduction in the flexural strength from 76.6 MPa (for S11) to 47.70 MPa (for S44). The results of the biodegradation investigation revealed that the higher content of PCL was accompanied by a higher weight loss of the blends over time. Also, applying the SEM imaging after testing the biodegradation revealed that the resultant structural changes (the formation of holes, cracks, and rough structure) are a consequence of biodegradation occurring. Additionally, the mechanical properties characterization of the prepared blends was reperformed subsequent to the biodegradation to evaluate the process effects on the blends. The results revealed that the mechanical characteristics deteriorated with the continuation of biodegradation, with percentages ranged from 1.03 to 8.87% for the tested properties compared to the original values before the biodegradation.

## Supplementary Information

Below is the link to the electronic supplementary material.

Supplementary Material 1

## Data Availability

All data generated or analyzed during this study are included in this published article and supplementary information files.
