# Supplementary material for: Characterization of doping polycaprolactone (PCL) and nano calcium carbonate (NCC) into polystyrene (PS) thermoplastic network
Source: Sci Rep. 2025 Nov 18;15:40397. doi: 10.1038/s41598-025-23821-2 (PMC12627763; doi:10.1038/s41598-025-23821-2)
Supplement: Supplementary file 1 — Supplementary Material 1 [file 41598_2025_23821_MOESM1_ESM.docx]

**Table S1:** The IR peaks and their positions for the as-synthesized PS-doped PCL/NCC blends

| Peak Position / Wavenumber (cm^-1^) | Assignments | The peak is attributed to |
| --- | --- | --- |
| 2926 | Asymmetric CH_2_ stretching | PS |
| 2852 | Asymmetric/Symmetric CH_2_ stretching | PS |
| 1449 | Asymmetric CH_3_ bending/stretching vibration of C–O | PS and/or NCC |
| 1602, 1492 | Presence of the benzene ring | PS |
| 3026, 906 | Aromatic C–H stretching | PS |
| 2940 | Asymmetric CH_2_ stretching | PCL |
| 2852 | Symmetric CH_2_ stretching | PCL |
| 1731 | Ester carbonyl C=O stretching | PCL |
| 1298 | C–O and C–C stretching in the crystalline phase | PCL |
| 1241 | Asymmetric C–O–C stretching | PCL |
| 1173 | Symmetric C–O–C stretching | PCL |
| 875, 712  1420 | Bending vibration of C–O  Stretching vibrations of C–O | NCC  NCC |
| 2516 | Combination of the three peaks 1420, 875, and 712 | NCC |
| 3455 | Stretching and asymmetric stretching vibration of O―H bond | NCC |
